# Vascular endothelial growth factor-A and Poly(A) binding protein-interacting protein 2 expression in human head and neck carcinomas: correlation and prognostic significance

**DOI:** 10.1038/sj.bjc.6603108

**Published:** 2006-04-25

**Authors:** C Onesto, J-M Hannoun-Lévi, E Chamorey, J-L Formento, A Ramaioli, G Pagès

**Affiliations:** 1 Institute of Signalling, Developmental Biology and Cancer Research, CNRS – UMR 6543, University of Nice Sophia-Antipolis (Equipe labellisée Ligue Nationale Contre le Cancer), 33 avenue de Valombrose, 06189 Nice Cedex, France; 2 Department of Radiation Oncology, Antoine Lacassagne anti-Cancer Centre, 33 avenue de Valombrose, 06189 Nice Cedex, France; 3 Department of statistics, Antoine Lacassagne anti-Cancer Centre, 33 avenue de Valombrose, 06189 Nice Cedex, France; 4 Department of Oncopharmacology, Antoine Lacassagne anti-Cancer Centre, 33 avenue de Valombrose, 06189 Nice Cedex, France

**Keywords:** vascular endothelial growth factor-A, Poly(A) binding protein-interacting protein 2, head and neck carcinoma, human tumour specimens, prognostic value

## Abstract

Vascular endothelial growth factor-A (VEGF-A) has been demonstrated to play an important role in tumour angiogenesis and to influence prognosis in many cancers. However its prognostic value in head and neck squamous cell carcinomas (HNSCCs) remains controversial. Therefore, we investigated the clinical relevance of VEGF-A expression in HNSCCs and analysed whether its expression was associated with PAIP2 protein levels, a VEGF-A mRNA-binding partner that strongly regulates VEGF-A expression in tissue culture. We determined the correlation of VEGF-A and PAIP2 protein levels, quantitatively evaluated in tumour tissue homogenates from 54 patients with HNSCC, to clinicopathological parameters. We showed that VEGF-A expression in HNSCC is correlated to the stage of tumour differentiation (*P*=0.050) and is an independent prognostic factor for progression-free survival (*P*=0.001) and overall survival (*P*=0.0004). In a pharynx carcinoma cell line, we demonstrated by RNA interference that VEGF-A expression is closely controlled by PAIP2. Moreover, in human HNSCCs, VEGF-A expression is significantly correlated to PAIP2 protein levels (*P*=0.0018). Nevertheless, PAIP2 expression is associated with neither clinicopathological factors nor patient's survival. Our data suggest that, in contrast to PAIP2 protein levels, which are unrelated to tumour prognosis, VEGF-A expression could serve as a prognostic marker in head and neck cancer and may be helpful for targeted therapies.

Head and neck cancer, including oral cancer, is the sixth most common type of human cancer, with an annual incidence of more than 500 000 cases worldwide. Smoking and alcohol abuse are the two major contributing factors of this disease. Head and neck squamous cell carcinoma (HNSCC) is the most common and the most aggressive type of head and neck tumour. Head and necksquamous cell carcinoma includes cancers arising from the oral cavity, pharynx (oro- and hypo-) and larynx. Despite major advances in cellular and molecular biology of cancer, as well as in therapeutic strategies, the survival rate for head and neck cancer, and particularly for HNSCC, has not improved in the last 30 years, with a 5-year overall survival rate of approximately 50% (Mao *et al*, 2004). Like most epithelial cancers, HNSCC develops through the accumulation of multiple genetic and epigenetic alterations, such as deletion of tumour suppressor regions, mutations, *de novo* promoter methylation of tumour suppressor genes, and amplification or overexpression of oncogenes (Mao *et al*, 2004). While early stage head and neck tumours can be successfully treated with surgery, the treatment of patients presented with an advanced stage using combined therapies (surgery followed or not by radiotherapy or radiochemotherapy alone) does not achieve good local control and overall survival. Development of multiple primary tumours, as well as tumour recurrences, are major causes of treatment failure. Moreover, there is no specific molecular marker currently used routinely to predict the clinical behaviour of head and neck cancer. In recent years, several studies have characterised changes in gene-expression in HNSCCs compared with normal oral mucosa. Some molecular markers have been identified and correlated with poor prognosis, such as p53, cyclin D1 or epidermal growth factor receptor (EGFR) (Hunter *et al*, 2005). Markers related to tumour neovascularisation could also predict the outcome in head and neck cancer patients. To grow beyond a microscopic size, most tumours must initiate angiogenesis (Folkman, 2002). One of the key angiogenic factors is the vascular endothelial growth factor-A (VEGF-A), which promotes tumour neovascularisation. In HNSCC, VEGF-A is considered to be a leading candidate of tumour angiogenesis, exhibiting its effect on the vasculature in paracrine and probably autocrine patterns (Kyzas *et al*, 2005b). Furthermore, VEGF-A has been described as an important prognostic factor in many types of human cancers (Dvorak, 2002). Several retrospective studies in HNSCC have demonstrated that VEGF-A expression is associated with clinicopathological factors and/or poor patient's outcome, suggesting that VEGF-A could serve as a prognostic marker (Eisma *et al*, 1997; Maeda *et al*, 1998; Mineta *et al*, 2000; Smith *et al*, 2000; Lim *et al*, 2003; Kimura *et al*, 2004; Uehara *et al*, 2004; Kyzas *et al*, 2005b). However, the status of VEGF-A remains unclear since other HNSCC retrospective studies have shown no correlation between VEGF-A expression and tumour prognosis (Salven *et al*, 1997; Tae *et al*, 2000; Do *et al*, 2004; Schimming *et al*, 2004). These conflicting results may be due partly to the method of detection of VEGF-A expression (ie immunohistochemistry), used in most of the previous reports, and which is a semiquantitative method. Therefore, we proposed to quantitatively evaluate the prognostic significance of VEGF-A protein levels in HNSCC, using an ELISA.

Moreover, we have recently demonstrated in several tumour cell lines that VEGF-A expression is highly regulated by an RNA-binding and stabilizing partner of the VEGF-A mRNA, the Poly(A) binding protein-interacting protein 2 (PAIP2) (Onesto *et al*, 2004). Thus, we investigated in the present study whether VEGF-A expression is associated with PAIP2 expression in HNSCC and examined whether PAIP2 expression is related to clinicopathological parameters and patient's survival.

## PATIENTS AND METHODS

### Patients

This retrospective study includes a total of 54 patients diagnosed with an HNSCC between January 2000 and March 2002 at the Antoine Lacassagne anti-Cancer Centre (Nice, France), and for whom both tumour specimens (obtained from the primary tumour and immediately frozen in liquid nitrogen after excision) and clinical outcomes were available. All patients were treated and followed-up at this same institution. All tumour specimens obtained with informed consent, come from either surgical excision during total tumour removal or endoscopic examination performed at the time of diagnosis. Hence, all patients were fresh cases and had not received any previous treatment. Patient characteristics are summarised in Table 1. Briefly, with a median follow-up of 25 months (1–62), this study analysed 54 patients (sex ratio M/F=2.4) with a median age at diagnosis of 57 (43–75) and 63 (29–86) for male and female, respectively. Anatomic sites were oral cavity (39%), pharynx (oro- and hypo-, 43%) and larynx (18%). Most of patients were classified T3–T4 (T1–T2 31%, T3–T4 69%), presented with positive cervical lymph nodes (N0 33%, N+ 67%) and tumour with high/moderate differentiation grade (high/moderate 60%, low 40%). For statistical analysis, we grouped T and N stages as follows: T1–T2 and T3–T4, as well as N0 (node negative) and N+ (=N1+N2+N3; node positive). Treatment policy for early stage tumours (T1–T2) was either surgery, associated or not with post-operative radiation therapy, or radiotherapy alone. Locally advanced stage tumours (T3–T4) were treated with concomitant regimen associating radiotherapy plus chemotherapy (total dose of 70 Gy in 35 fractions over 7 weeks+5FU 750 mg m^−2^ d1-5 cycle 1, thereafter 430 mg m^−2^ d1-5 cycles 2 and 3 associated with CDDP 100 mg m^−2^ d1 of each cycle) (Calais *et al*, 1999). Patients presenting with cervical lymph node involvement after surgery received preferentially a postoperative radiotherapy, associated sometimes with concomitant chemotherapy (especially for patients treated after 2001, for whom several of them have been included in a clinical trial (Bernier *et al*, 2004)). For each patient, post-treatment surveillance was performed every 3 months. Clinical and endoscopic exams were achieved alternatively by head and neck surgeon and radiation oncologist. CT-scans associated or not with magnetic resonance imaging of the cervical area were performed at the time of each clinical examination during the first year post-treatment and every 6 months afterwards. Bone scan, chest, abdominal or cerebral CT-scans were performed in case of distant metastatic disease suspicion.

### Cell line and culture conditions

The human pharynx carcinoma cell line Detroit 562 was from American Type Culture Collection. Cells were grown in Dulbecco's modified Eagle's medium (DMEM) (Life Technologies, Inc.) supplemented with 7.5% heat-inactivated fetal calf serum.

### Preparation of cytosolic extracts from tumour tissues

Tumour specimens immediately frozen after surgical excision were mechanically pulverised in liquid nitrogen. The resulting powders were homogenised with a Polytron PT-1020 in Tris-HCl buffer, pH 7.4, containing EDTA 1 mM, dithiothreitol 0.5 mM, sodium molybdate 10 mM and inhibitors of proteases and phosphatases and centrifuged at 105 000 × **g** for 1 h to obtain cytosols that were stored at −80°C until analysis for PAIP2 and VEGF-A expression levels.

### RNA interference experiments

The following 21-mer oligoribonucleotides and their reverse sequence were synthesised by Eurogentec (Liege, Belgium). Four independent small_interfering RNA (siRNAs) were designed in different regions of human PAIP2 complete cDNA (NCB # Accession NM_016480). The respective sequences are the following:

PAIP2 (1): 5′-GGCUCUUCUCUGGAAGAUCTT-3′;
PAIP2 (2): 5′-GAUCUUGUGGUCAAGAGCATT-3′;
PAIP2 (3): 5′-UCAUGAAGAUGACAAUCCATT-3′
PAIP2 (4): 5′-CGACAACCAACAUCAGCCATT-3′.

The siRNA sequence targeting the EGFP coding sequence (NCB # Accession AF323988) was used as an irrelevant siRNA and is the following: 5′-GAACGGCAUCAAGGUGAACTT-3′.

Detroit 562 cells were transiently transfected with siRNA as previously described (Onesto *et al*, 2004). At 48 h following the second transfection, cells were lysed and analysed either by Western blotting for PAIP2 expression or by Northern blotting for VEGF-A mRNA expression. In parallel, tissue culture supernatants were collected and tested by immunoassay for secreted VEGF-A expression.

### Northern blotting

Cells were lysed in Trizol Reagent buffer (GIBCO BRL, Cergy-Pentoise, France). RNA was prepared according to the manufacturer's protocol. RNA (20) *μ*g was used for Northern blotting analysis of PAIP2 and VEGF-A mRNA expression as previously described (Onesto *et al*, 2004).

### Western blotting

50 *μ*g of whole cell extracts from Detroit 562 cells or cytosolic extracts from tumour homogenates were subjected to Western blotting using a homemade polyclonal anti-PAIP2 antibody (1/2000) (Onesto *et al*, 2004). Bound antibody was detected using the CDP-star system (New England Biolabs, Beverly, MA, USA).

The expression levels of *β*-actin or extracellular signals-regulated kinase 2 (ERK2), used as loading controls, have also been monitored using a monoclonal anti-*β*-actin antibody (1/8000) (clone AC-15, Sigma, Saint-Quentin-Fallaviers, France) or a polyclonal anti-ERK2 antibody (1/5000) (homemade), respectively.

The level of PAIP2 for each tumour sample was quantified with a GeneGnome chemiluminescent imaging system (Syngene, Frederick, MD, USA) and normalised to the signal for *β*-actin as well as to the signal for PAIP2 in HeLa cell extracts run in parallel on each blot. The latter represents an internal control which rules out any variation between the different blots.

### ELISA assay

VEGF-A concentration of cytosolic extracts from tumour specimens and of tissue culture supernatants were determined using the Quantikine Human VEGF-A Immunoassay system, following the manufacturer's guidelines (R&D Systems, Lille, France).

### Statistical analysis

The statistical significance relationship between expression of VEGF-A or PAIP2 and clinicopathological parameters was assessed using *χ*^2^ test confirmed by Fisher's exact test. Correlation between VEGF-A and PAIP2 expression was examined using the Pearson's test confirmed by Spearman rank test. Overall survival was defined as the time between diagnosis and death due to any cause (in our study, only one patient died from another cause than HNSCC cancer). Progression-free survival was defined as the time between diagnosis and the first clinical or pathological evidence of local or distant recurrent disease or patient death. Refractory cancers under treatment were considered to be in progression at the date of diagnosis. Metastatic-free survival was defined as the time between diagnosis and the first clinical or pathological evidence of metastatic recurrent disease. Probabilities of progression-free and overall survival were estimated using the Kaplan–Meier method and differences were analysed using the log-rank test. The role of each possible prognostic factor and their combined effects in predicting progression-free and overall survival were analysed using the Cox proportional hazards survival model. Data were considered statistically significant if *P*-values were less than or equal to 0.05. All statistical tests were two-sided. For statistical evaluation, the R 2.0.1 software was used (Ihaka and Gentleman, 1996).

## RESULTS

### VEGF-A expression and analysis of the correlation to clinicopathological factors

We determined the level of VEGF-A expression, by immunoassay, in cytosols from 54 human head and neck tumours. We observed that VEGF-A is expressed in nearly all tumours (98.3%). Median VEGF-A is 830 pg·mg^−1^ of protein (undetectable to 7285 pg·mg^−1^ of protein).

For evaluation of HNSCC, several clinicopathological parameters were used, such as the clinical stage, the presence of lymph node metastases (nodal status) and the degree of tumour differentiation (differentiation stage). Using univariate analysis, we investigated the correlation between VEGF-A expression and these established clinicopathological tumour parameters. As shown in Table 2, VEGF-A expression significantly correlates with the tumour differentiation stage (*P*=0.050). Tumours with poor differentiation express higher VEGF-A levels (⩾median) than highly differentiated tumours. No significant association between the clinical stage or nodal status and VEGF-A expression in tumour tissues was found (Table 2).

### VEGF-A expression is an independent prognostic factor for progression-free survival and overall survival

To investigate whether VEGF-A expression was associated with the outcome of patients with HNSCC in our study, survival analysis were performed on progression-free and overall survival of the 54 patients. In total 26 patients (48%) experienced disease recurrence and 35 (65%) died during the follow-up period. It is noteworthy that patients who exhibited high VEGF-A levels (⩾median) had a significantly higher rate of disease recurrence (*P*=0.014) and death (*P*=0.0006) (data not shown).

**Figure 1:**
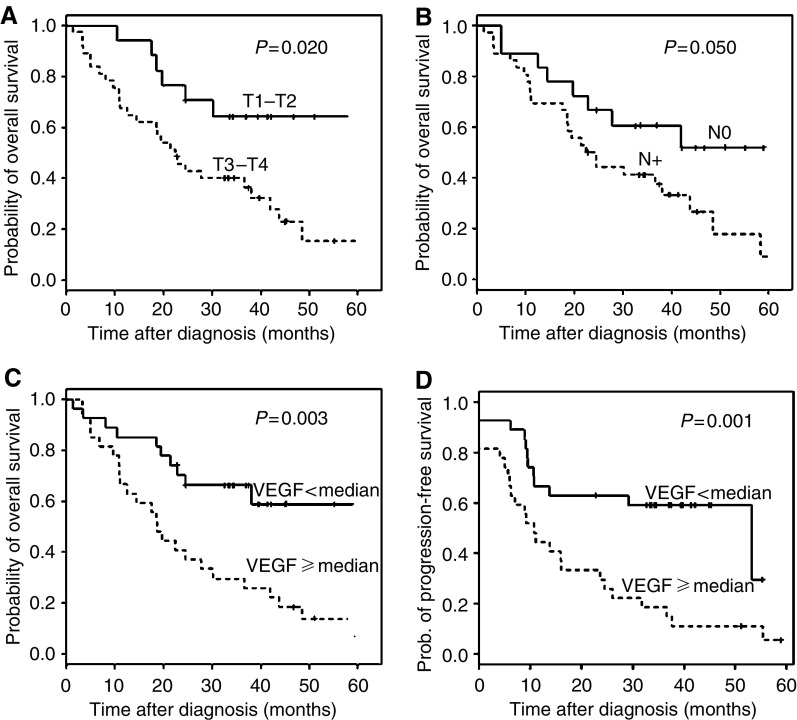
Univariate survival analysis investigating the impact of clinical stage (**A**), nodal status (**B**) or VEGF-A expression (**C**) on overall survival of patients with head and neck carcinomas. Graph D illustrates the impact of VEGF-A expression on progression-free survival.

Table 3 and Figure 1C and D show in univariate survival analysis, that VEGF-A expression is a factor of poor prognosis for progression-free survival (*P*=0.001), as well as for overall survival (*P*=0.003). Interestingly, VEGF-A expression appears as a prognostic factor of metastatic-free survival (*P*=0.002) but not of local recurrence-free survival (*P*=0.37) (data not shown). Furthermore, the clinical stage and nodal status, both known as poor prognostic factors for patient's outcome, are, in our study, also significantly correlated to overall survival (*P*=0.020 and 0.050, respectively; Table 3, Figure 1A and B). The clinical stage also correlates to progression-free survival (*P*=0.03, Table 3) and patient age is another prognostic parameter of progression-free and overall survival (Table 3).

The prognostic significance of VEGF-A expression, patient age, clinical stage or nodal status, in term of progression-free and overall survival, were then analysed in a multivariate Cox regression model (Table 4). VEGF-A expression is identified as an independent prognostic parameter for both progression-free survival and overall survival (*P*=0.001 and 0.0004, respectively; Table 4). Similar results are obtained for clinical stage on progression-free and overall survivals (*P*=0.01 and 0.002, respectively; Table 4). We also found that patient age has an independent prognostic impact on progression-free survival (*P*=0.004; Table 4). Conversely, inclusion of neither patient age nor nodal status in the overall survival analysis, added significant independent prognostic information (*P*=0.08 and 0.13, respectively, Table 4).

### PAIP2 strongly regulates VEGF-A expression in a human pharynx carcinoma cell line

In a previous study, we demonstrated that the Poly(A) binding protein-Interacting Protein 2 (PAIP2) is a strong regulator of VEGF-A mRNA expression in a Chinese hamster fibroblast-derived cell line as well as in two different human tumour cell lines derived from epidermoid carcinoma (A-431) and cervix adenocarcinoma (HeLa) (Onesto *et al*, 2004). By interacting with the 3′untranslated region of VEGF-A mRNA, PAIP2 stabilises this mRNA, leading to an increase in VEGF-A expression (Onesto *et al*, 2004).

Prior to analysis for a potential correlation between PAIP2 and VEGF-A expression in the 54 HNSCC specimens, we first checked whether the effect of PAIP2 on VEGF-A expression also occurs in a head and neck carcinoma cell line. To this end, we silenced PAIP2 expression by RNA interference in Detroit 562 cells derived from a human pharynx carcinoma. It is noteworthy that these cells secrete higher VEGF-A levels than those secreted by HeLa and A-431 cells (Detroit 562 cells: 7100±1410 pg of VEGF-A·ml·1·10^−6^ cells (*n*=3); HeLa cells: 3776±781 pg·ml·1·10^−6^ cells (*n*=3) and A-431 cells: 6242±1539 pg·ml·1·10^−6^ cells (*n*=2)).

**Figure 2:**
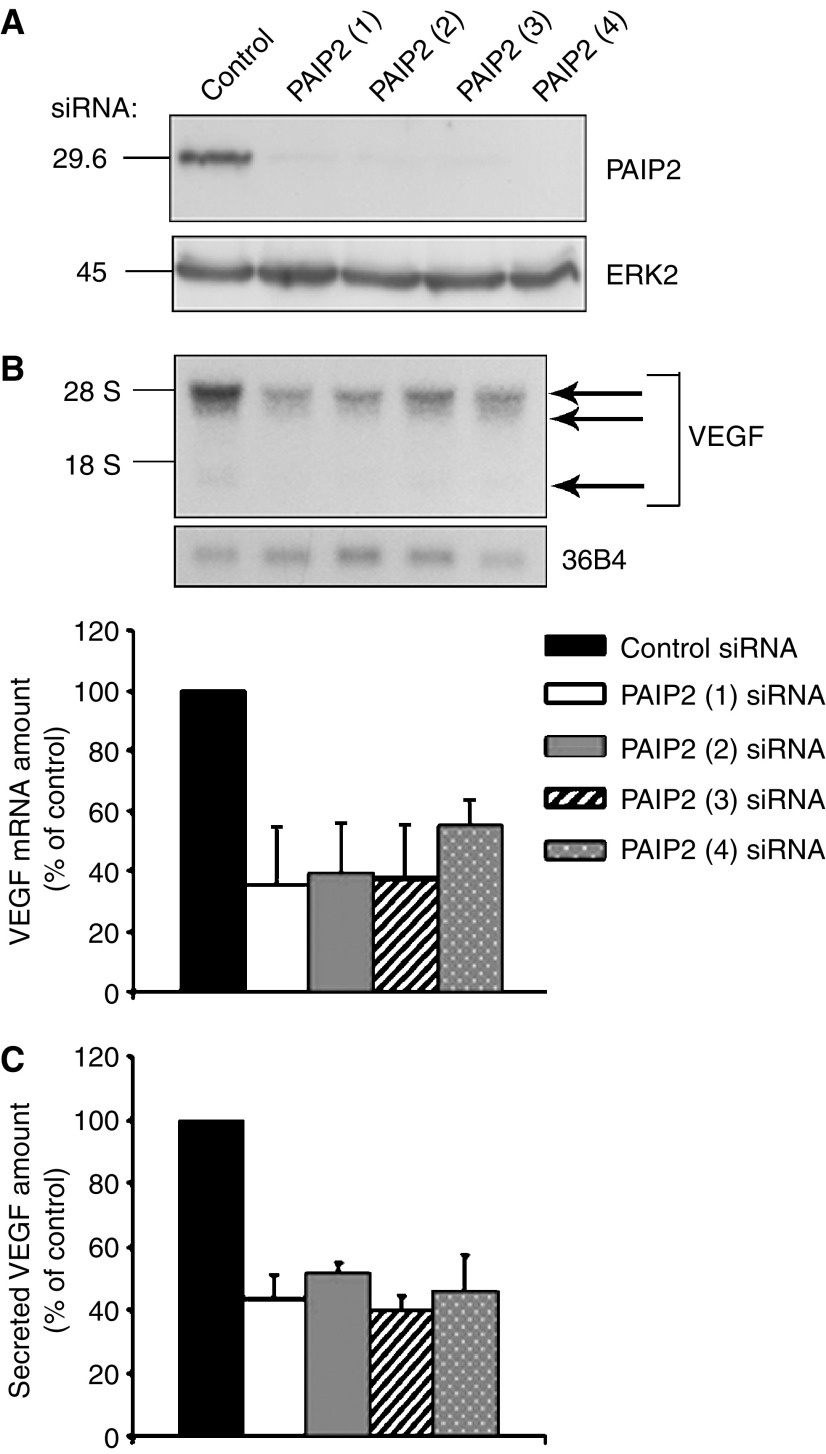
Silencing of PAIP2 by RNA interference inhibits VEGF-A expression in a human pharynx carcinoma cell line. (**A**) Total protein extracts from Detroit 562 cells transiently transfected twice with siRNAs against PAIP2 or GFP, were analysed by Western blotting using a polyclonal anti-PAIP2 antibody. The amounts of ERK2 are shown as a loading control. (**B**) Total RNA extracted from the same cells was subjected to Northern blotting analysis for VEGF-A mRNA expression (upper panel). The arrows point to the different VEGF-A isoforms. 36B4 RNA is shown as a loading control. The levels of VEGF-A mRNA were quantified by phosphorImaging analysis (lower panel). The values are normalised to 36B4 RNA. Values obtained with the control siRNA are taken as 100%. The data shown represent the means and standard errors of two independent experiments. (**C**) Tissue culture supernatants from the same cells were analysed using a human VEGF-A specific ELISA. The values are normalised to the cell number. Values obtained with the control siRNA are taken as 100%. The data shown represent the means and standard errors of two independent experiments each performed in triplicate.

Detroit 562 cells were transfected twice with four different PAIP2-targeting siRNAs or an irrelevant control siRNA, and analysed 48 h following the second transfection. In contrast to the unchanged level of ERK2 used as a loading control, PAIP2 is efficiently depleted by siRNA (a mean of 96% of inhibition), whatever the specific-siRNA sequence used (Figure 2A). Moreover, in conditions where PAIP2 is specifically silenced by siRNA, expression of the VEGF-A mRNA is strongly reduced, as illustrated in Figure 2B, upper panel. Quantification of these results shows an approximate 60% decrease of VEGF-A mRNA levels in cells treated with PAIP2 siRNA sequences 1–3 and a 46% reduction with PAIP2 siRNA sequence 4 (Figure 2B, lower panel). Furthermore, as shown in Figure 2C, the PAIP2-mediated decrease in VEGF-A expression not only occurs at the mRNA level but also at the protein level with an approximate 50% inhibition of secreted VEGF-A when PAIP2 is silenced. Hence, PAIP2 strongly regulates VEGF-A expression in a head and neck carcinoma cell line.

### Positive correlation between PAIP2 and VEGF-A expression in human head and neck tumour specimens

We determined PAIP2 expression by Western blotting in cytosols from the 54 HNSCC specimens. Poly (A) binding protein-interacting proteing 2 is present in nearly all tumours (98.3%) and is expressed at various levels in the different tumour specimens (data not shown). Median PAIP2 is 0.712 arbitrary units (a.u.), (undetectable to 2.470 a.u).

We then investigated whether PAIP2 expression is associated with VEGF-A expression in the human tumour samples. We found a statistically significant positive correlation between PAIP2 and VEGF-A expression (*P*=0.0018 – data not shown).

By univariate analysis, we investigated the correlation between PAIP2 expression and clinicopathological tumour parameters. Concerning the clinical stage, 59% of the T1–T2 patients (*n*=17) show high PAIP2 levels (⩾median) *vs* 49% of the T3–T4 patients (*n*=34). For nodal status, high levels of PAIP2 (⩾median) are expressed in 50% of the patients without nodal metastases (*n*=18) *vs* 53% of node positive patients (*n*=36). Concerning the differentiation stage, high PAIP2 levels (⩾median) are distributed as follows: high (14 patients – 57%), moderate (18 patients – 56%) and low (21 patients – 43%). No statistical significance was found between all these clinicopathological variables and the degree of PAIP2 expression in tumour tissues (data not shown).

Similar statistically insignificant results were obtained between PAIP2 levels and patient's progression-free survival (*P*=0.80) or overall survival (*P*=0.59) (data not shown). Hence, despite its strong correlation with VEGF-A protein levels, PAIP2 expression is neither a prognostic factor for tumour progression nor for patient's outcome in the 54 human HNSCC analyzed.

## DISCUSSION

In this study, we investigated in HNSCC the expression pattern and prognostic significance of VEGF-A levels as well as levels of a VEGF-A regulatory protein, the Poly(A) binding protein-Interacting Protein 2 (PAIP2).

We have shown that VEGF-A expression is significantly associated with the tumour differentiation stage, poorly differentiated tumours expressing higher VEGF-A levels than highly differentiated tumours. In a recently published meta-analysis in HNSCCs, Kyzas *et al* (2005a) have described a trend towards a correlation of VEGF positivity with poor histological differentiation. Our study provides the conclusive proof that there is a strong correlation between VEGF-A expression and tumour differentiation in head and neck carcinomas. Prior retrospective studies, except for the recent meta-analysis (Kyzas *et al*, 2005a), did not show any correlation between those two parameters (Salven *et al*, 1997; Maeda *et al*, 1998; Mineta *et al*, 2000; Tae *et al*, 2000; Do *et al*, 2004; Kyzas *et al*, 2005b). The observed difference between all the studies could be explained by a number of factors, including heterogeneity in head and neck tumour populations, biased selection of patients and method of detection of VEGF-A protein levels, the latter being one of the most critical aspect in this type of retrospective study. It should be noted that in our study, we measured VEGF-A expression using a quantitative method (ie ELISA), in contrast to all previous reports (except one (Mineta *et al*, 2000)), in which VEGF-A protein levels have only been measured by a semiquantitative evaluation method (i.e. immunohistochemistry). A similar variability in findings between different studies is also observed for the association of VEGF-A expression with clinical stage or nodal status. Some reports (Salven *et al*, 1997; Maeda *et al*, 1998; Neuchrist *et al*, 1999; Do *et al*, 2004), including our study, show no correlation between VEGF-A protein levels and these clinicopathological parameters, whereas others reported a relationship (Eisma *et al*, 1999; Mineta *et al*, 2000; Tae *et al*, 2000; Lim *et al*, 2003). These conflicting results highlight the necessity to perform retrospective studies using reliable methodology (preferentially quantitative) and a large cohort of patients.

Consistent with other findings (Eisma *et al*, 1997; Eisma *et al*, 1999; Mineta *et al*, 2000, 2002; Smith *et al*, 2000; Kyzas *et al*, 2005a, 2005b), we showed that high VEGF-A protein levels (⩾median) predicted a higher rate of disease recurrence and shorter progression-free interval. Moreover, tumours expressing high VEGF-A levels are also more likely to recur distantly than tumours with low VEGF-A levels (<median). Multivariate analysis identified VEGF-A expression as an independent prognostic factor for progression-free survival and distant recurrence-free survival. Several studies have shown that VEGF-A, as well as VEGF-C, are upregulated in head and neck cancers, thus stimulating proliferation of vascular and lymphatic endothelial cells, and increasing vessel permeability (Rogers *et al*, 2005). The enhanced angiogenic activity could sustain growth of the primary tumour, potentiate dissemination and also support the establishment of micrometastases. This is consistent with the strong association observed in this study between VEGF-A expression and distant recurrence.

We also demonstrated that VEGF-A protein levels are closely associated to overall survival. Subsequently, multivariate analysis showed that VEGF-A expression is, in our study, an independent prognostic factor for overall survival. Two other studies have reported similar results: patients with tumours expressing high levels of VEGF-A have a shorter overall survival than those with tumours expressing low VEGF-A levels, and the impact of VEGF-A is not a combined effect with other markers of poor prognosis (Smith *et al*, 2000; Lim *et al*, 2003). Taken together, the results of VEGF-A implication on distant recurrence-free and overall survival support the hypothesis that high VEGF-A levels predict aggressive disease.

Vascular endothelial growth factor-A expression is tightly controlled, its regulation occurring at several levels of gene expression: transcription, mRNA stability and translation. We previously identified a new protein partner of the VEGF-A mRNA, the latter being a labile mRNA. This new VEGF-A mRNA-binding protein, called PAIP2, stabilises VEGF-A mRNA, thus contributing to an increase in VEGF-A secretion (Onesto *et al*, 2004). The significant association observed in this study between VEGF-A and PAIP2 protein levels in human head and neck carcinomas supports the data we obtained in several tumour cell lines (Onesto *et al*, 2004), including a pharynx carcinoma cell line (this report). It also suggests that VEGF-A expression could be closely controlled by PAIP2 in human tumour tissue. Interestingly, VEGF-A expression is also correlated to PAIP2 protein levels in human breast cancer specimens (Onesto C., unpublished results), bringing forward the importance of PAIP2 in regulating VEGF-A expression in human cancers. It would be interesting to compare PAIP2 and VEGF-A protein levels measured in malignant tissues to their normal homologues, in order to determine whether they also vary in parallel in a normal context. Increased expression of VEGF-A has been demonstrated in HNSCC cell lines, xenografts and clinical specimens (Rogers *et al*, 2005). We could imagine that any dysregulation of PAIP2 expression could explain the abnormal VEGF-A protein levels in a tumour context. Thus, the regulation of VEGF-A overexpression in malignant head and neck carcinomas may provide an interesting example for a possible contribution of dysregulation of mRNA stability to the progression of cancer. However, in contrast to VEGF-A, PAIP2 expression is not associated to patient's outcome. Thus, despite its significant influence on VEGF-A expression in HNSCC, PAIP2 is insufficient to confer to VEGF-A its impact on poor prognosis. Hence, PAIP2 is probably not the only protein that regulates VEGF-A expression in HNSCC. We hypothesise that other factors, which also regulate VEGF-A expression, will be responsible of VEGF-A impact on poor prognosis. It would be informative to also analyse the potential correlation of VEGF-A status with the expression of HuR, another VEGF-A mRNA-stabilising protein (Levy *et al*, 1998), which has also been described in different types of human cancers to be related to poor tumour prognosis (Denkert *et al*, 2004a, 2004b). Other elements, particularly implicated in tumorigenesis and considered as prognostic factors in HNSCC may also play a role in VEGF-A regulation, such as the hypoxia-inducible factor (HIF)-1 alpha, the product of the tumour suppressor gene *p53*, the Epidermal Growth Factor Receptor (EGFR) or the cyclooxygenase-2 (COX-2) (Quon *et al*, 2001; Cohen, 2004). Furthermore, strong correlations between these proteins and VEGF-A expression have been identified in head and neck cancers (O-Charoenrat *et al*, 2000; Gallo *et al*, 2001; Lim *et al*, 2003; Kimura *et al*, 2004). It would be interesting to analyse possible associations of these molecular markers with VEGF-A status in our study.

The results of this study, coupled with the existing literature, suggest that patients with tumours with high VEGF-A protein levels may be at a greater risk of poor progression-free survival, poor overall survival and metastatic disease. Hence, the VEGF-A status could emerge as an important factor in establishing prognosis and selecting treatment modalities for HNSCC. Antiangiogenic targeted therapies may positively impact on treatment of HNSCC. At present, bevacizumab, a humanised monoclonal antibody that targets VEGF-A, is undergoing clinical evaluation (in association with EGFR-targeted drugs) in HNSCC (Caponigro *et al*, 2005). In the future, it would be helpful in clinical trials to determine whether VEGF-A could serve not only as a prognostic marker in head and neck carcinomas, but also as a predictive tool for the most efficient response to treatment.

## Figures and Tables

**Table 1:** Characteristics of 54 patients with head and neck squamous cell carcinomas

**Characteristics**	**#**	**%**
Male	38	70
Female	16	30

*Median age at diagnosis (y)*		
Male	57 (43–75)	
Female	63 (29–86)	

Median follow-up (mo)	25 (1–62)	
*Anatomic sites*		
Oral cavity	21	39
Oropharynx	14	26
Hypopharynx	9	17
Larynx	10	18

*Clinical stage*		
Tl	4	7
T2	13	24
T3	12	22
T4	25	47

*Nodal status*		
NO	18	33
N1	4	7
N2	29	54
N3	3	6

*Differentiation stage* a		
High	14	26
Moderate	18	34
Low	21	40

*Treatment*		
S	14	26
RT	5	9
CT+RT	16	30
S+RT	10	18
S+RT+CT	9	17

a For one patient the information was not available.

y: years, mo: months, CT: chemotherapy, RT: radiotherapy, S: surgery.

**Table 2:** Univariate analysis of VEGF-A expression and the various clinicopathological factors

		**VEGF-A**		
**Variable**	**Number of patients**	**<Median**	**⩾Median**	***P*-value**
*Clinical stage*				NS
T1–T2	17	8 (47%)	9 (53%)	
T3–T4	37	19 (51%)	18 (49%)	

*Nodal stage*				NS
N0	18	8 (44%)	10 (56%)	
N+	36	19 (53%)	17 (47%)	

*Differentiation stage*				0.05
High	14	9 (64%)	5 (36%)	
Moderate	18	9 (50%)	9 (50%)	
Low	21	8 (38%)	13 (62%)	

NS: not significant; VEGF-A: Vascular endothelial growth factor A.

**Table 3:** Univariate analysis (Kaplan–Meier) on progression-free survival and overall survival of all patients according to patient age, clinical stage, nodal status and VEGF-A expression

	**Progression-free survival**				**Overall survival**			
**Variable**	**Nb of patients**	**Nb of events^a^**	**Median survival time (mo)**	***P*-value**	**Nb of patients**	**Nb of events^a^**	**Median survival time (mo)**	***P*-value**
*Patient age*				<1 × 10^−3^				0.030
<70 years	47	30	26.1		47	29	36.7	
⩾70 years	7	7	6.0		7	6	8.2	

*Clinical stage*				0.03				0.020
T1–T2	17	9	55.4		17	7	58.4	
T3–T4	37	28	10.8		37	28	22.4	

*Nodal status*				NS				0.050
N0	18	10	30.7		18	8	Not reached	
N+	36	27	13.8		36	27	23.4	

*VEGF-A expression*				0.001				0.003
<median	27	12	53.2		27	11	62.3	
⩾Median	27	25	10.7		27	24	18.7	

a Event for progression-free survival corresponds to the onset of local or distant recurrent disease. Event for overall survival corresponds to patient's death.

mo: months, Nb: number, NS: not significant; VEGF-A, Vascular endothelial growth factor A.

**Table 4:** Multivariate analysis (Cox model) on progression-free survival and overall survival of all patients

	**Progression-free survival**			**Overall survival**		
**Variable**	**RR**	**95% CI of RR**	***P*-value**	**RR**	**95% CI of RR**	***P*-value**
Patient age <70 years/⩾70years	4.40	(1.75–11.08)	0.004	—	—	NS
Clinical stage (T1–T2/T3–T4)	2.65	(1.17–6.0)	0.012	3.44	(1.45–8.17)	0.002
Nodal status (N0/N+)	—	—	—	—	—	NS
VEGF-A expression (</⩾ median)	3.16	(1.54–6.48)	0.001	3.61	(1.70–7.65)	0.0004

RR: relative risk, CI: confidence interval, NS: not significant; VEGF, Vascular endothelial growth factor A.
